# "Targeted disruption of the epithelial-barrier by *Helicobacter pylori*"

**DOI:** 10.1186/1478-811X-9-29

**Published:** 2011-11-01

**Authors:** Lydia E Wroblewski, Richard M Peek

**Affiliations:** 1 Division of Gastroenterology, Department of Medicine, Vanderbilt University Medical Center, Nashville, TN 37232, USA; 2 Department of Cancer Biology, Vanderbilt University Medical Center, Nashville, TN 37232, USA; 3 Department of Veterans Affairs Medical Center, Nashville, TN 37212, USA

## Abstract

*Helicobacter pylori *colonizes the human gastric epithelium and induces chronic gastritis, which can lead to gastric cancer. Through cell-cell contacts the gastric epithelium forms a barrier to protect underlying tissue from pathogenic bacteria; however, *H. pylori *have evolved numerous strategies to perturb the integrity of the gastric barrier. In this review, we summarize recent research into the mechanisms through which *H. pylori *disrupts intercellular junctions and disrupts the gastric epithelial barrier.

## Review

### The gastric epithelium and *Helicobacter pylori*

The gastric epithelium is comprised of a single layer of cells that invaginate to form highly organized gastric glands, populated by a distinct variety of cell types. The gastric epithelium can mediate digestive processes; however, an essential function of the gastric mucosal epithelium is to maintain a protective barrier that separates luminal contents containing pathogenic microorganisms such as *Helicobacter pylori*, from the underlying tissue compartments. *H. pylori *is a Gram-negative bacterial pathogen that selectively colonizes the gastric epithelium of approximately half of the world's population [1]. The most common outcome of *H. pylori *infection is chronic asymptomatic gastritis [2]; however, long-term colonization with *H. pylori *significantly increases the risk of developing gastro-duodenal diseases. Among infected individuals, approximately 10% develop peptic ulcer disease, 1-3% develop gastric adenocarcinoma, and less than 0.1% develop mucosa associated lymphoid tissue (MALT) lymphoma [3]. Accordingly, *H. pylori *is classified as a Type I carcinogen, and is considered to be the most common etiologic agent of infection-related cancers, which represent 5.5% of the global cancer burden [4].

*H. pylori *strains are extremely diverse and have evolved sophisticated virulence strategies that affect host cell signaling pathways and play an important role in determining the outcome of infection [1]. Disease-associated *H. pylori *strains possess the *cag *pathogenicity island (*cag *PAI), which encodes components of a bacterial type IV secretion apparatus, and functions to export the terminal product of the *cag *PAI, CagA, across the bacterial membrane and into host gastric epithelial cells [5-7]. There are two mechanisms reported through which *H. pylori *may translocate CagA into host cells. One mechanism requires the interaction of CagL, a pilus localized component of the type IV secretion apparatus, with integrin α_5_β_1 _on host epithelial cells [8]. An alternative mechanism is the type IV secretion apparatus induces externalization of phosphatidylserine, which resides on the inner leaflet of the cell membrane under resting conditions. CagA is then able to interact with phosphatidylserine and gain entry to host epithelial cells [9]. Although all *H. pylori *strains induce gastritis, strains that contain the *cag *PAI (*cag*^+^) augment the risk for severe gastritis, atrophic gastritis, and distal gastric cancer compared to those strains that lack the *cag *island (*cag*^-^) [10-21]. Following injection into host epithelial cells, CagA becomes tyrosine phosphorylated at glutamate-proline-isoleucine-tyrosine-alanine (EPIYA) motifs, which induces cell morphological changes, initially termed the 'hummingbird phenotype'. These alterations are linked to cellular migration and, importantly, may compromise the integrity of the gastric barrier [22-26]. Non-phosphorylated CagA also exerts effects within gastric epithelial cells that contribute to pathogenesis; including, but not limited to, activation of β-catenin, disruption of apical-junctional complexes, and loss of cellular-polarity [27-32]. Non-phosphorylated CagA interacts with the cell adhesion protein E-cadherin, the hepatocyte growth factor receptor c-Met, phospholipase PLC-g, the adaptor protein Grb2, and the kinase PAR1b/MARK2 [30,32-34], which culminate in pro-inflammatory and mitogenic responses, disruption of cell-cell junctions, and loss of cell polarity. These events will be discussed in more detail in subsequent sections (see sections: Disruption of the tight junction by *H. pylori *and Disruption of the adherens junction by *H. pylori*).

### Intercellular junctions

**Figure 1:**
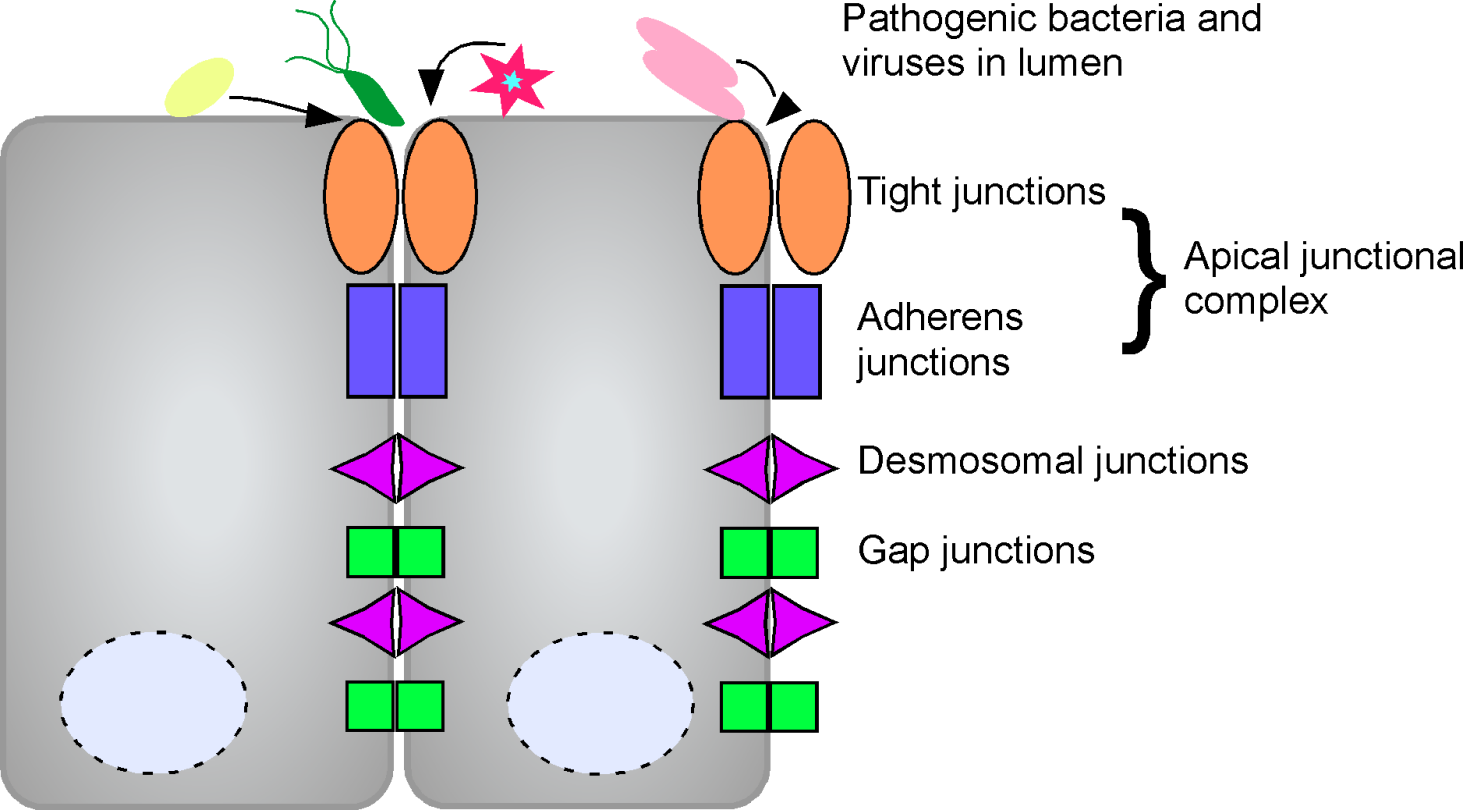
**Intercellular junctions form the epithelial barrier**. Several bacteria, including *H. pylori*, and viruses interact with and disrupt cell-cell junctions of polarized epithelia. Intercellular junctions include tight junctions, adherens junctions, desmosomal junctions, and gap junctions.

Intercellular contacts are required to maintain the molecular architecture and selective barrier function of epithelial tissue. Within the gastric mucosa, barrier function is essential for preventing potentially harmful elements present in the gastric lumen from gaining access to the gastric mucosa. Intercellular junctions include the tight-junction which is juxtaposed at the most apical region of polarized cells, and the adherens junction which is located immediately below; collectively, these comprise the apical junctional complex which plays a pivotal role in regulating paracellular flux of ions and small molecules. The apical junctional complex also maintains cell polarity and regulates cell proliferative processes through relatively undefined signaling pathways. In addition to the apical junctional complex, gap junctions and desmosomes are also constituents which contribute to cell-cell contacts (Figure 1). In contrast to the apical junctional complex, which forms a tight seal between epithelial cells, gap junctions link the cytosol of adjacent cells to permit ions and small molecules to shuttle between cells [35]. Little is known in regard to how *H. pylori *may alter gap junctions, although there are data to suggest that CagA-positive strains may down-regulate gap junctions [36]. Desmosomes tightly tether adjacent cells through attachment to intermediate filaments [37], and loss of desmosomes has recently been linked to tumor development and early invasion [38,39]. To our knowledge, there are no reports of *H. pylori *interacting with desmosomes, making this an attractive area of study. What is clear, however, is that *H. pylori *preferentially adhere to gastric epithelial cells in close proximity to the apical junctional complex [27,40], and can alter localization of component proteins that constitute apical-junctional complexes [27,41-43]. Furthermore, barrier function is compromised in *H. pylori*-induced gastritis [44], and disruption of the apical junctional complex is associated with gastric cancer [45].

### Overview of tight junctions

**Figure 2:**
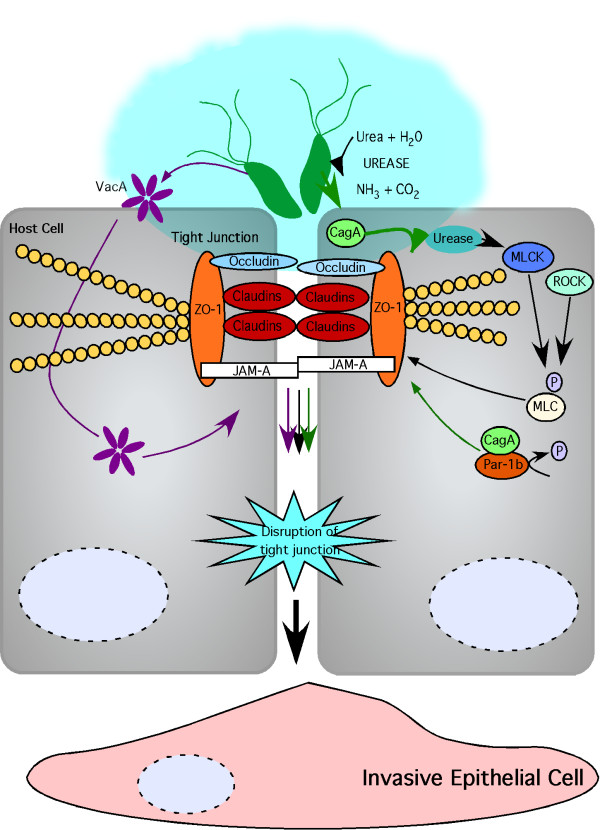
**Dysregulation of the tight junction by *H. pylori***. *H. pylori* preferentially bind in close proximity to the tight junction and disrupt gastric barrier function, cell adhesion, and cell polarity which culminates in an invasive phenotype. Tight junctions are composed of the integral membrane proteins occludin, claudins, and junctional adhesion molecule (JAM)-A, as well as zonula occludens-1 (ZO-1). Tight junction function is disrupted by urease activity and phosphorylation of myosin light chain (MLC) by myosin light chain kinase (MLCK) or Rho kinase (ROCK). Translocated CagA interacts with partitioning-defective 1 (PAR1) to inhibit phosphorylation by blocking PAR1 kinase activity and disrupts the tight junction. VacA also increases tight junction permeability.

Tight junctions are located at the most apical region of the cell; they mediate adhesion between epithelial cells, and form tight seals between cells to create the major barrier in the paracellular pathway. Tight junctions are highly dynamic structures consisting of integral membrane proteins and membrane-associated proteins, which collectively form a complex protein network. Scaffolding proteins link transmembrane proteins to the actin cytoskeleton. Integral membrane proteins, such as occludin, claudins, and junctional adhesion molecules (JAMs) are important components of the tight junction that span junctions and connect membranes on adjacent cells to form a seal (Figure 2). Collectively, these components play critical roles in maintenance of barrier function, cell polarity, and intercellular adhesion.

Occludin was the first transmembrane tight junction protein to be identified [46], and it contains four transmembrane domains, two extracellular loops, and two intracellular loops. The C-terminus physically associates with ZO-1 and this interaction is essential for tight junction assembly [47]. Occludin deficient mice exhibit a complex phenotype, and initial studies indicated that occludin was not required for tight junction assembly or maintenance of barrier function [48]. However, subsequent characterization of occludin deficient mice suggests that occludin is essential for regulation of epithelial tight junctions. Occludin is highly phosphorylated on serine and threonine residues and phosphorylated occludin is the form that is associated with the tight junction [49]. Recent work suggests PKCη and PKCζ phosphorylation of occludin is required for complete assembly of the tight junction [50,51].

Claudins represent a family of 24 transmembrane proteins and are the main constituents of the tight junction intercellular strands [45]. Claudins, like occludin, are tetraspanning proteins with two extracellular loops and two intracellular loops; however, they do not posses sequence homology to occludin. Claudins mediate calcium-independent cell-cell adhesion and form either homodimers or heterodimers. Different combinations of claudin isoforms can mediate cell-type-specific differences in tight junctions [45].

JAM-A is a member of the immunoglobulin superfamily of proteins and contains an extracellular domain comprised of two Ig-like domains, a single transmembrane domain, and a short cytoplasmic C-terminal domain with a PDZ binding motif that is important for the interaction with tight junction scaffolding proteins. The extracellular domain of JAM-A contains dimerization motifs and forms homophilic contacts. The detailed role of JAM-A in regulating tight junction function is not fully understood; however, since it is known to interact with many other proteins, JAM-A may regulate tight junction formation by targeting proteins to the tight junction and may regulate epithelial permeability, inflammation, proliferation and migration [52,53]. Dimerization of JAM-A is required for the assembly of a protein complex with the PDZ domain-containing molecules Afadin and PDZ-guanine nucleotide exchange factor (GEF). This activates Rap1A, which stabilizes β1 integrin protein levels and increases cell migration [53]. JAM-A also acts as a receptor for viruses and is required for hematogenous dissemination of reovirus [54]. Whether JAM-A is utilized as a receptor by bacteria is currently unknown.

In addition to integral membrane proteins, tight junction proteins also include membrane-associated proteins such as zonula occludens-1 (ZO-1). ZO-1 is a member of the MAGUK (membrane-associated guanylate kinase homologs) family, characterized by a PDZ domain, SH3 domain and guanylate kinase domain. ZO-1 interacts with the C-terminus of occludin [55] and with claudins [56], and can also interact with proteins found in the adherens junction [57] and attach to the actin cytoskeleton [58].

### Disruption of the tight junction by *H. pylori*

Disruption of the tight junction complex is associated with a variety of human diseases and cancers, including cancers of the gastrointestinal tract [45]. *H. pylori *are commonly found adhering to gastric epithelial cells, preferentially in close proximity to the apical junctional complex [27,40,59], possibly to gain maximal access to essential nutrients released by gastric epithelial cells [60]. Viable *H. pylori *have also been identified within the lamina propria, gastric lymph nodes, and within the intracellular canaliculi of parietal cells [61-63]; thus, an alternative hypothesis is that *H. pylori *may utilize the tight junction as a means to gain entry to the lamina propria [64].

Numerous studies have demonstrated that *H. pylori *modulates the tight junction [27,29,41-43,65-68]; however, what is less clear are the specific *H. pylori *constituents that mediate these changes in barrier function. In studies using polarized MDCK cells infected with a variant of *H. pylori *that was cell-adapted for increased adhesion, translocated CagA was shown to recruit ZO-1 and JAM-A to the site of bacterial attachment [27]. In MDCK cells, ectopic expression of GFP-CagA was also shown to disrupt the tight junction by inducing mis-localization of ZO-1 to the basolateral membrane, and inducing loss of apicobasal polarity characterized by a redistribution of the apical glycoprotein gp135 to the basolateral membrane and adoption of an invasive cellular phenotype [29]. Concordant with studies using MDCK cells, co-culture of primary human gastric epithelial cells results in membrane disruption of ZO-1 and redistribution of ZO-1 to small vesicles in the cytoplasm. However, the precise role of CagA in this cascade remains to be fully determined as total levels of ZO-1 protein remain unchanged between uninfected cells and those infected with CagA-positive or CagA-negative strains [42].

CagA has also been shown to dysregulate the tight junction through an interaction with partitioning-defective 1b (PAR1b)/microtubule affinity-regulating kinase 2 (MARK2). PAR1b is one of four structurally related members of the PAR1 family of kinases, and has an essential role in maintaining epithelial cell polarity by phosphorylating microtubule-associated proteins (MAPs), and destabilizing microtubules to permit the asymmetric distribution of molecules required for cells to maintain polarity [32,69-71]. CagA binds all four PAR1 isoforms with varying affinity [72], and the PAR1b-binding region of CagA has been identified as the 16-amino-acid CagA sequence also termed the CagA-multimerization (CM) sequence, which is involved in CagA dimerization [73]. The initial 14 amino acids of the CM motif bind to the MARK2 kinase substrate binding site, thereby mimicking a host cell substrate [74] to inactivate the kinase activity of PAR1, leading to defects in epithelial cell polarity and disruption of tight junctions [32] (Figure 2). Interestingly, the number of CM repeats correlates with the virulence potential of CagA. Within Western *H. pylori *strains, the number of CagA CM repeats is directly proportional to the ability of CagA to bind PAR1b, while the CM sequence of CagA isolated from East-Asian *H. pylori *strains binds PAR1b more strongly than the CM sequence isolated from Western strains of *H. pylori *[75]. There is also a direct correlation between the level of PAR1b-binding-activity of CagA and the extent of cellular morphologic aberrations or disruption of the tight junction [75].

In other studies, CagA-independent alterations in tight junction structure and function have been demonstrated. The addition of purified VacA to MDCK cells lowers transepithelial electrical resistance (TER) and increases tight junction permeability to low-molecular weight molecules and ions. However, purified VacA-induced changes in tight junction function were not associated with alterations in ZO-1, occludin, or the adherens junction protein E-cadherin [76]. This was confirmed using live bacterial infection of MDCK cells with an isogenic *vacA *mutant strain. In this system, no alterations were seen in TER over a 20 hour infection [68]. In contrast, co-culture of MKN28 gastric epithelial cells with an isogenic *vacA *mutant strain decreased TER to the same extent as wild-type *H. pylori *[43]. We speculate that these reported differences in the role of VacA on modulating TER may be due to using different cell models and/or different strains of *H. pylori*. It would be interesting to determine *in vivo *if VacA is required for gastric barrier disruption.

In two independent studies, *H. pylori *strain SS1 was reported to disrupt barrier function in the gastric mucosa [41,66]. These findings also suggest that CagA is not important for *H. pylori *disruption of the tight junction, because although *H. pylori *strain SS1 is CagA positive, it lacks a functional type IV secretion system and cannot inject CagA into epithelial cells [77]. Another research group used canine intestinal epithelial cells, and found that co-culture of these cells with *H. pylori *stain SS1 induces redistribution of claudin-4 and claudin-5 and decreases membrane expression of these two tight junction proteins. Interestingly, the distribution and expression of ZO-1 and JAM-A were not changed [41]. More recently, the *H. pylori *Cag^+ ^strain 60190 was found to disrupt claudin-4 localization, and decrease cellular expression of claudin-4 in a CagA- and VacA-independent manner [78]. Further dissection of the signaling pathways involved suggested that *H. pylori *phosphorylates IL-1 receptor type I, and in a Rho kinase-dependent manner disrupts claudin-4 at the tight junction [78].

The influence of *H. pylori *generated ammonium on tight junctions has also been investigated. Ammonium produced by *H. pylori *reduces TER in Caco-2 human colonic epithelial cells, which is associated with increased levels of a 42 kDa truncated form of occludin [67]. Urease catalyzes the hydrolysis of urea into carbon dioxide and ammonia, and functional urease activity was found to be required for *H. pylori*-induced disruption of TER in gastric epithelial cells [43] (Figure 2).

Paracellular permeability controlled by the tight junction can be regulated by myosin light chain kinase (MLCK)-mediated phosphorylation of myosin light chain (MLC), which increases the tension placed on the tight junction [79]. In SCBN canine intestinal cells it was determined using a selective inhibitor of MLCK, that activation of MLCK by *H. pylori *strain SS1 leads to decreased barrier function and increased expression of claudin-4 and claudin-5 [41]. Collectively these data suggest that in a CagA-independent manner, *H. pylori *decreases expression of claudin-4 and claudin-5, activates MLCK and subsequently disrupts barrier function [41]. In another study using a membrane-permeable inhibitor of MLCK (PIK) [80], activation of MLCK by *H. pylori *and the subsequent phosphorylation of MLC were also shown to disrupt barrier function by decreasing TER in human gastric epithelial cells, and *ureB *was required for maximal phosphorylation of MLC [43]. PKC activation may also be important for *H. pylori*-regulation of the tight junction [65] as activation of PKC increases TER by reducing phosphorylation of MLC [81] and decreased TER in T84 colonic epithelial cells induced by *H. pylori *was prevented by concurrent activation of PKC using the phorbol ester phorbol 12-myristate 13-acetate (PMA) [65].

Several studies have shown that *H. pylori *disrupts occludin localization at the tight junction [41,43,66]. This has been observed in two different cell line models [41,43], as well as in two different mouse models of *H. pylori *infection [43,66]. Despite the consistency in results between models, the *H. pylori *virulence factor required for disruption of occludin remains to be determined. The precise role of occludin in regulating barrier function is currently unclear, although, occludin is implicated in regulation of gastric barrier function [82], and emerging evidence suggests an important role for occludin in mediating barrier permeability.

Alterations in tight junction proteins induced by *H. pylori *and the virulence factors that are important for this disruption appear to be strain specific and discrepancies between different research groups are likely confounded by the use of different model systems. Another factor that may contribute to discrepancies as to the role of CagA in disrupting the tight junction may be the polarization state of the cells under study [60,83]. Recent work examining the role of CagA for replication of *H. pylori *on MDCK cells has shown CagA-dependent as well as CagA-independent effects, depending on the polarization state of the host cell. CagA is required for *H. pylori *to disrupt MDCK cell polarity, and CagA-deficient *H. pylori *are not able to replicate on polarized cells when they are unable to access nutrients from the basolateral surface [60].

### Adherens junction

**Figure 3:**
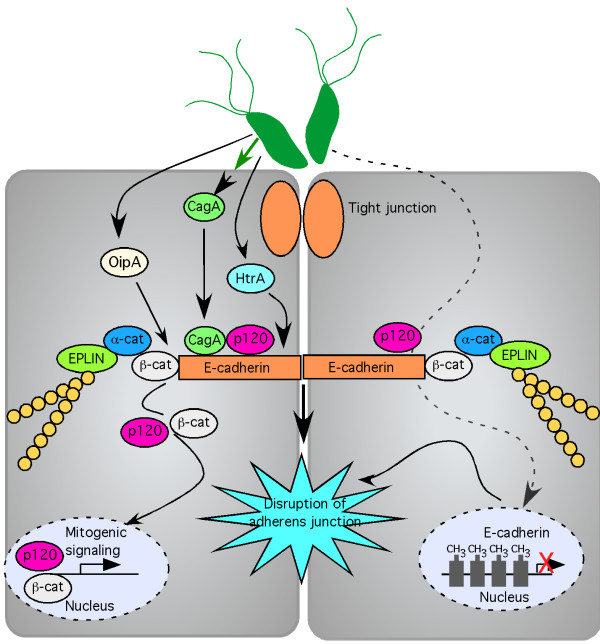
**Dysregulation of the adherens junction by *H. pylori***. *H. pylori*-translocated CagA interacts with E-cadherin and p120. This destabilizes the adherens junction and results in nuclear translocation of β-catenin and p120 and alterations in transcriptional activity. The *H. pylori *outer membrane protein OipA disrupts adherens junctions through redistribution of β-catenin, and *H. pylori*-secreted high-temperature requirement A (HtrA) cleaves E-cadherin, disrupting the adherens junction. Hypermethylation of the E-cadherin promoter also occurs in response to *H. pylori *infection and epithelial protein lost in neoplasm (EPLIN) binds α-catenin and links the cadherin-catenin complex with actin.

Adherens junctions are required for maintenance of adhesive cell-cell contacts, cell polarity, and for signal transduction to the nucleus to regulate transcription. Adherens junctions are dynamic structures and are formed on a foundation of calcium-dependent homophilic contacts between E-cadherin on the surface of adjacent epithelial cells [84]. Other key components of the adherens junction are the armadillo protein family members p120-catenin (p120) and β-catenin, and the actin-binding protein α-catenin. E-cadherin has long extracellular and cytoplasmic domains; the extracellular domains of E-cadherin form homophilic interactions [85], while the cytoplasmic tail interacts directly with several intracellular proteins including p120 and β-catenin, which in turn bind α-catenin [86-88]. Previous data suggested that α-catenin interacts directly with the actin cytoskeleton; however this has been called into question as the interactions between β-α-catenin and α-catenin-actin were not found to occur simultaneously *in vitro *[89,90]. More recently EPLIN (epithelial protein lost in neoplasm) was identified as an α-catenin binding partner, and EPLIN was determined to mediate the interaction of the cadherin-catenin complex with actin [91] (Figure 3). There are currently no published reports as to whether *H. pylori *may disrupt the adherens junction through interactions with EPLIN, making this a potentially fruitful area of study.

### Disruption of the adherens junction by *H. pylori*

In numerous studies, *H. pylori *infection has been shown to induce E-cadherin gene promoter methylation, which ultimately leads to a reduction in E-cadherin expression [92-94]. Loss of E-cadherin function is associated with gastric cancer [92-94], and hypermethylation of the E-cadherin promoter can be reversed by eradication of *H. pylori *[93-95]. Decreasing the stability of the adherens junction by altering E-cadherin expression may be one mechanism through which *H. pylori *disrupts gastric barrier function and promotes disease progression (Figure 3).

*H. pylori *infection disrupts the adherens junction and initiates translocation of E-cadherin, β-catenin, and p120 from the membrane into the cytoplasm of epithelial cells [31,96-98]. Specifically, transfected CagA physically interacts with E-cadherin in a manner that does not require CagA tyrosine phosphorylation [30]. The interaction of CagA with E-cadherin results in destabilization of the E-cadherin/β-catenin complex, and accumulation of cytoplasmic and nuclear β-catenin, which subsequently transactivates β-catenin-dependent genes that may promote carcinogenesis [30,99] (Figure 3). It is now thought that CagA not only interacts with E-cadherin, but also interacts with p120, and forms a multiprotein complex composed of c-Met, E-cadherin, and p120. This prevents tyrosine phosphorylation of c-Met and p120, and attenuates the invasive phenotype induced by CagA [99]. Through activation of PI3-K/Akt signaling by non-phosphorylated CagA, *H. pylori *also activates β-catenin and downstream pathways associated with disease development [100]

Under normal physiological conditions, cytoplasmic β-catenin is regulated by glycogen synthase kinase-3β (GSK-3β), which phosphorylates β-catenin within a multi-protein inhibitory complex that includes the adenomatous polyposis coli (APC) tumor suppressor protein. This complex constitutively targets β-catenin for degradation by the ubiquitin-proteasome pathway [101]. However, in gastric adenocarcinoma along with other cancers, increased expression of β-catenin, mutations within APC, and/or inhibition of GSK-3β are frequently observed, all of which function to stabilize β-catenin in the cytoplasm [102]. Other mechanisms through which *H. pylori *induces increased cytoplasmic expression of β-catenin are via PI3K-dependent inactivation of GSK-3β [100,103], and direct interaction with membrane associated β-catenin via CagA [30,104]. Cytoplasmic β-catenin subsequently translocates to the nucleus where it interacts with T-cell factor/lymphoid enhancer factor-1 (Tcf/LEF-1) transcription factors to regulate transcription of genes that can influence carcinogenesis [30,104]. In a gerbil model of infection, nuclear accumulation of β-catenin occurs following infection with carcinogenic Cag^+ ^*H. pylori *strains [28]. Concordantly, in human gastric biopsies there is an increase in levels of nuclear β-catenin in gastric epithelium harvested from patients infected with *H. pylori cag^+ ^*strains when compared to persons infected with *H. pylori cag^- ^*strains, or uninfected persons [28]. Recent work has shed new light on the role of CagA in disrupting the adherens junction with the discovery of an inhibitory domain within the N-terminus of CagA [105]. The first 200 amino acids of the CagA N-terminus counteract host responses evoked by the C-terminus of CagA and reduce host-cell responses by strengthening cell-cell contacts and decreasing CagA-induced β-catenin activity [105]. Thus it appears that CagA has evolved domains to tightly regulate β-catenin activation within host cells.

Although important, CagA is not the only bacterial factor that disrupts adherens junction proteins [97,106-108]. In a Mongolian gerbil model of gastric cancer, inactivation of the *H. pylori *outer membrane protein OipA decreased nuclear localization of β-catenin and reduced the incidence of gastric cancer, suggesting OipA may be associated with the redistribution of β-catenin and promotion of the carcinogenic process [106]. Proteolytic cleavage of E-cadherin is independent of CagA in studies that utilized a human breast cancer cell (MCF-7) model [97], and in human gastric NCI-N87 cells [109]. Recent work has identified *H. pylori *high-temperature requirement A (HtrA) as a novel secreted virulence factor that cleaves E-cadherin and disrupts the adherens junction [107], (Figure 3). Loss of E-cadherin from the adherens junction is also associated with dissociation of β-catenin and p120 from the adherens junction into the cytosol. Similar to findings by Bebb *et al*. [108], β-catenin did not translocate to the nucleus, and as such, did not modulate transcription [97].

Under normal physiological conditions, nuclear expression of p120 is low; however, in tumor cells, expression of p120 is elevated [110-112]. *H. pylori *has recently been associated with mislocalization of p120 to the nucleus in human gastric epithelia, and in infected murine primary gastric epithelial cells [42,98]. Further analysis of downstream signaling pathways determined that p120 mis-localized to the nucleus in response to *H. pylori *acts to relieve transcriptional repression of *mmp-7*, a matrix metalloproteinase implicated in gastric tumorogenesis, by an interaction with Kaiso [98]. Nagy *et al*. have also recently reported that a p120- and β-catenin target gene, PPARδ, regulates gastric epithelial proliferation via activation of cyclin E. These are potentially important mechanisms through which *H. pylori *may lower the threshold for developing gastric cancer [98].

## Conclusions

The gastric epithelium is primed to secrete effector molecules that control gastric function, and the highly organized nature of gastric glands is essential for regulating gastric integrity and maintaining a protective barrier between harmful luminal contents and the underlying tissue compartments. *H. pylori *has developed numerous strategies to penetrate the gastric epithelial barrier by altering the structure and function of the apical junctional complex. The role of CagA in disrupting the apical junction complex is divisive; however, the actions of CagA are critical in a number of contexts. In addition to CagA, *H. pylori *also utilizes other factors to modify the gastric barrier. These include VacA, OipA, urease, and the newly identified HtrA, in addition to disrupting the gastric barrier through altering cell polarity. Future studies will provide further insight into understanding how *H. pylori *factors and signaling pathways culminate in loss of barrier function. These studies are of utmost importance as many gastric diseases including gastric cancer may develop as a result of compromised barrier function.

## Competing interests

The authors declare that they have no competing interests.

## Authors' contributions

LEW and RMP drafted and wrote the manuscript. LW prepared the figures. Both authors read and approved the final manuscript

## References

[B1] Wroblewski LE Peek RM Jr Wilson KT Helicobacter pylori and gastric cancer: factors that modulate disease risk Clin Microbiol Rev 2010 23 713 739 10.1128/CMR.00011-10 PMC2952980 20930071

[B2] Peek RM Jr Blaser MJ Helicobacter pylori and gastrointestinal tract adenocarcinomas Nat Rev Cancer 2002 2 28 37 10.1038/nrc703 11902583

[B3] Peek RM Jr Crabtree JE Helicobacter infection and gastric neoplasia J Pathol 2006 208 233 248 10.1002/path.1868 16362989

[B4] Parkin DM Bray F Ferlay J Pisani P Global cancer statistics, 2002 CA Cancer J Clin 2005 55 74 108 10.3322/canjclin.55.2.74 15761078

[B5] Covacci A Rappuoli R Tyrosine-phosphorylated bacterial proteins: Trojan horses for the host cell J Exp Med 2000 191 587 592 10.1084/jem.191.4.587 PMC2195833 10684850

[B6] Censini S Lange C Xiang Z Crabtree JE Ghiara P Borodovsky M Rappuoli R Covacci A cag, a pathogenicity island of Helicobacter pylori, encodes type I-specific and disease-associated virulence factors Proc Natl Acad Sci USA 1996 93 14648 14653 10.1073/pnas.93.25.14648 PMC26189 8962108

[B7] Akopyants NS Clifton SW Kersulyte D Crabtree JE Youree BE Reece CA Bukanov NO Drazek ES Roe BA Berg DE Analyses of the cag pathogenicity island of Helicobacter pylori Mol Microbiol 1998 28 37 53 10.1046/j.1365-2958.1998.00770.x 9593295

[B8] Kwok T Zabler D Urman S Rohde M Hartig R Wessler S Misselwitz R Berger J Sewald N Konig W Backert S Helicobacter exploits integrin for type IV secretion and kinase activation Nature 2007 449 862 866 10.1038/nature06187 17943123

[B9] Murata-Kamiya N Kikuchi K Hayashi T Higashi H Hatakeyama M Helicobacter pylori exploits host membrane phosphatidylserine for delivery, localization, and pathophysiological action of the CagA oncoprotein Cell Host Microbe 2010 7 399 411 10.1016/j.chom.2010.04.005 20478541

[B10] Blaser MJ Chyou PH Nomura A Age at establishment of Helicobacter pylori infection and gastric carcinoma, gastric ulcer, and duodenal ulcer risk Cancer Res 1995 55 562 565 7834625

[B11] Cover TL Dooley CP Blaser MJ Characterization of and human serologic response to proteins in Helicobacter pylori broth culture supernatants with vacuolizing cytotoxin activity Infect Immun 1990 58 603 610 10.1128/iai.58.3.603-610.1990 PMC258508 2307514

[B12] Crabtree JE Taylor JD Wyatt JI Heatley RV Shallcross TM Tompkins DS Rathbone BJ Mucosal IgA recognition of Helicobacter pylori 120 kDa protein, peptic ulceration, and gastric pathology Lancet 1991 338 332 335 10.1016/0140-6736(91)90477-7 1677696

[B13] Crabtree JE Wyatt JI Sobala GM Miller G Tompkins DS Primrose JN Morgan AG Systemic and mucosal humoral responses to Helicobacter pylori in gastric cancer Gut 1993 34 1339 1343 10.1136/gut.34.10.1339 PMC1374537 8244098

[B14] Kuipers EJ Perez-Perez GI Meuwissen SG Blaser MJ Helicobacter pylori and atrophic gastritis: importance of the cagA status J Natl Cancer Inst 1995 87 1777 1780 10.1093/jnci/87.23.1777 7473834

[B15] Parsonnet J Friedman GD Orentreich N Vogelman H Risk for gastric cancer in people with CagA positive or CagA negative Helicobacter pylori infection Gut 1997 40 297 301 10.1136/gut.40.3.297 PMC1027076 9135515

[B16] Peek RM Jr Miller GG Tham KT Perez-Perez GI Cover TL Atherton JC Dunn GD Blaser MJ Detection of Helicobacter pylori gene expression in human gastric mucosa J Clin Microbiol 1995 33 28 32 10.1128/jcm.33.1.28-32.1995 PMC227873 7699060

[B17] Queiroz DM Mendes EN Rocha GA Oliveira AM Oliveira CA Magalhaes PP Moura SB Cabral MM Nogueira AM cagA-positive Helicobacter pylori and risk for developing gastric carcinoma in Brazil Int J Cancer 1998 78 135 139 10.1002/(sici)1097-0215(19981005)78:2<135::aid-ijc1>3.0.co;2-# 9754640

[B18] Rudi J Kolb C Maiwald M Zuna I von Herbay A Galle PR Stremmel W Serum antibodies against Helicobacter pylori proteins VacA and CagA are associated with increased risk for gastric adenocarcinoma Dig Dis Sci 1997 42 1652 1659 10.1023/a:1018849112533 9286230

[B19] Shimoyama T Fukuda S Tanaka M Mikami T Munakata A Crabtree JE CagA seropositivity associated with development of gastric cancer in a Japanese population J Clin Pathol 1998 51 225 228 10.1136/jcp.51.3.225 PMC500644 9659265

[B20] Torres J Perez-Perez GI Leal-Herrera Y Munoz O Infection with CagA+ Helicobacter pylori strains as a possible predictor of risk in the development of gastric adenocarcinoma in Mexico Int J Cancer 1998 78 298 300 10.1002/(SICI)1097-0215(19981029)78:3<298::AID-IJC6>3.0.CO;2-Q 9766561

[B21] Vorobjova T Nilsson I Kull K Maaroos HI Covacci A Wadstrom T Uibo R CagA protein seropositivity in a random sample of adult population and gastric cancer patients in Estonia Eur J Gastroenterol Hepatol 1998 10 41 46 10.1097/00042737-199801000-00008 9512952

[B22] Segal ED Cha J Lo J Falkow S Tompkins LS Altered states: involvement of phosphorylated CagA in the induction of host cellular growth changes by Helicobacter pylori Proc Natl Acad Sci USA 1999 96 14559 14564 10.1073/pnas.96.25.14559 PMC24475 10588744

[B23] Asahi M Azuma T Ito S Ito Y Suto H Nagai Y Tsubokawa M Tohyama Y Maeda S Omata M Helicobacter pylori CagA protein can be tyrosine phosphorylated in gastric epithelial cells J Exp Med 2000 191 593 602 10.1084/jem.191.4.593 PMC2195829 10684851

[B24] Stein M Rappuoli R Covacci A Tyrosine phosphorylation of the Helicobacter pylori CagA antigen after cag-driven host cell translocation Proc Natl Acad Sci USA 2000 97 1263 1268 10.1073/pnas.97.3.1263 PMC15590 10655519

[B25] Odenbreit S Puls J Sedlmaier B Gerland E Fischer W Haas R Translocation of Helicobacter pylori CagA into gastric epithelial cells by type IV secretion Science 2000 287 1497 1500 10.1126/science.287.5457.1497 10688800

[B26] Stein M Bagnoli F Halenbeck R Rappuoli R Fantl WJ Covacci A c-Src/Lyn kinases activate Helicobacter pylori CagA through tyrosine phosphorylation of the EPIYA motifs Mol Microbiol 2002 43 971 980 10.1046/j.1365-2958.2002.02781.x 11929545

[B27] Amieva MR Vogelmann R Covacci A Tompkins LS Nelson WJ Falkow S Disruption of the epithelial apical-junctional complex by Helicobacter pylori CagA Science 2003 300 1430 1434 10.1126/science.1081919 PMC3369828 12775840

[B28] Franco AT Israel DA Washington MK Krishna U Fox JG Rogers AB Neish AS Collier-Hyams L Perez-Perez GI Hatakeyama M Activation of beta-catenin by carcinogenic Helicobacter pylori Proc Natl Acad Sci USA 2005 102 10646 10651 10.1073/pnas.0504927102 PMC1180811 16027366

[B29] Bagnoli F Buti L Tompkins L Covacci A Amieva MR Helicobacter pylori CagA induces a transition from polarized to invasive phenotypes in MDCK cells Proc Natl Acad Sci USA 2005 102 16339 16344 10.1073/pnas.0502598102 PMC1274241 16258069

[B30] Murata-Kamiya N Kurashima Y Teishikata Y Yamahashi Y Saito Y Higashi H Aburatani H Akiyama T Peek RM Jr Azuma T Hatakeyama M Helicobacter pylori CagA interacts with E-cadherin and deregulates the beta-catenin signal that promotes intestinal transdifferentiation in gastric epithelial cells Oncogene 2007 26 4617 4626 10.1038/sj.onc.1210251 17237808

[B31] Suzuki M Mimuro H Suzuki T Park M Yamamoto T Sasakawa C Interaction of CagA with Crk plays an important role in Helicobacter pylori-induced loss of gastric epithelial cell adhesion J Exp Med 2005 202 1235 1247 10.1084/jem.20051027 PMC2213224 16275761

[B32] Saadat I Higashi H Obuse C Umeda M Murata-Kamiya N Saito Y Lu H Ohnishi N Azuma T Suzuki A Helicobacter pylori CagA targets PAR1/MARK kinase to disrupt epithelial cell polarity Nature 2007 447 330 333 10.1038/nature05765 17507984

[B33] Mimuro H Suzuki T Tanaka J Asahi M Haas R Sasakawa C Grb2 is a key mediator of helicobacter pylori CagA protein activities Mol Cell 2002 10 745 755 10.1016/s1097-2765(02)00681-0 12419219

[B34] Churin Y Al-Ghoul L Kepp O Meyer TF Birchmeier W Naumann M Helicobacter pylori CagA protein targets the c-Met receptor and enhances the motogenic response J Cell Biol 2003 161 249 255 10.1083/jcb.200208039 PMC2172921 12719469

[B35] Steinberg TH Gap junction function: the messenger and the message Am J Pathol 1998 152 851 854 PMC1858255 9546343

[B36] Tao R Hu MF Lou JT Lei YL Effects of H pylori infection on gap-junctional intercellular communication and proliferation of gastric epithelial cells in vitro World J Gastroenterol 2007 13 5497 5500 10.3748/wjg.v13.i41.5497 PMC4171286 17907295

[B37] Delva E Tucker DK Kowalczyk AP The desmosome Cold Spring Harb Perspect Biol 2009 1 a002543 10.1101/cshperspect.a002543 PMC2742091 20066089

[B38] Chun MG Hanahan D Genetic deletion of the desmosomal component desmoplakin promotes tumor microinvasion in a mouse model of pancreatic neuroendocrine carcinogenesis PLoS Genet 2010 6 10.1371/journal.pgen.1001120 PMC2940733 20862307

[B39] Beaudry VG Jiang D Dusek RL Park EJ Knezevich S Ridd K Vogel H Bastian BC Attardi LD Loss of the p53/p63 regulated desmosomal protein Perp promotes tumorigenesis PLoS Genet 2010 6 e1001168 10.1371/journal.pgen.1001168 PMC2958815 20975948

[B40] Hazell SL Lee A Brady L Hennessy W Campylobacter pyloridis and gastritis: association with intercellular spaces and adaptation to an environment of mucus as important factors in colonization of the gastric epithelium J Infect Dis 1986 153 658 663 10.1093/infdis/153.4.658 3950447

[B41] Fedwick JP Lapointe TK Meddings JB Sherman PM Buret AG Helicobacter pylori activates myosin light-chain kinase to disrupt claudin-4 and claudin-5 and increase epithelial permeability Infect Immun 2005 73 7844 7852 10.1128/IAI.73.12.7844-7852.2005 PMC1307049 16299274

[B42] Krueger S Hundertmark T Kuester D Kalinski T Peitz U Roessner A Helicobacter pylori alters the distribution of ZO-1 and p120ctn in primary human gastric epithelial cells Pathol Res Pract 2007 203 433 444 10.1016/j.prp.2007.04.003 17509776

[B43] Wroblewski LE Shen L Ogden S Romero-Gallo J Lapierre LA Israel DA Turner JR Peek RM Jr Helicobacter pylori dysregulation of gastric epithelial tight junctions by urease-mediated myosin II activation Gastroenterology 2009 136 236 246 10.1053/j.gastro.2008.10.011 PMC2678540 18996125

[B44] Sun YQ Soderholm JD Petersson F Borch K Long-standing gastric mucosal barrier dysfunction in Helicobacter pylori-induced gastritis in mongolian gerbils Helicobacter 2004 9 217 227 10.1111/j.1083-4389.2004.00227.x 15165257

[B45] Turner JR Molecular basis of epithelial barrier regulation: from basic mechanisms to clinical application Am J Pathol 2006 169 1901 1909 10.2353/ajpath.2006.060681 PMC1762492 17148655

[B46] Furuse M Hirase T Itoh M Nagafuchi A Yonemura S Tsukita S Occludin: a novel integral membrane protein localizing at tight junctions J Cell Biol 1993 123 1777 1788 10.1083/jcb.123.6.1777 PMC2290891 8276896

[B47] Mandell KJ Parkos CA The JAM family of proteins Adv Drug Deliv Rev 2005 57 857 867 10.1016/j.addr.2005.01.005 15820556

[B48] Saitou M Ando-Akatsuka Y Itoh M Furuse M Inazawa J Fujimoto K Tsukita S Mammalian occludin in epithelial cells: its expression and subcellular distribution Eur J Cell Biol 1997 73 222 231 9243183

[B49] Rao RK Basuroy S Rao VU Karnaky KJ Jr Gupta A Tyrosine phosphorylation and dissociation of occludin-ZO-1 and E-cadherin-beta-catenin complexes from the cytoskeleton by oxidative stress Biochem J 2002 368 471 481 10.1042/BJ20011804 PMC1222996 12169098

[B50] Suzuki T Elias BC Seth A Shen L Turner JR Giorgianni F Desiderio D Guntaka R Rao R PKC eta regulates occludin phosphorylation and epithelial tight junction integrity Proc Natl Acad Sci USA 2009 106 61 66 10.1073/pnas.0802741106 PMC2629239 19114660

[B51] Jain S Suzuki T Seth A Samak G Rao R PKCzeta phosphorylates occludin and promotes assembly of epithelial tight junctions Biochem J 2011 10.1042/BJ20110587 PMC3408004 21545357

[B52] Laukoetter MG Nava P Lee WY Severson EA Capaldo CT Babbin BA Williams IR Koval M Peatman E Campbell JA JAM-A regulates permeability and inflammation in the intestine in vivo J Exp Med 2007 204 3067 3076 10.1084/jem.20071416 PMC2150975 18039951

[B53] Severson EA Lee WY Capaldo CT Nusrat A Parkos CA Junctional adhesion molecule A interacts with Afadin and PDZ-GEF2 to activate Rap1A, regulate beta1 integrin levels, and enhance cell migration Mol Biol Cell 2009 20 1916 1925 10.1091/mbc.E08-10-1014 PMC2663925 19176753

[B54] Antar AA Konopka JL Campbell JA Henry RA Perdigoto AL Carter BD Pozzi A Abel TW Dermody TS Junctional adhesion molecule-A is required for hematogenous dissemination of reovirus Cell Host Microbe 2009 5 59 71 10.1016/j.chom.2008.12.001 PMC2642927 19154988

[B55] Muller SL Portwich M Schmidt A Utepbergenov DI Huber O Blasig IE Krause G The tight junction protein occludin and the adherens junction protein alpha-catenin share a common interaction mechanism with ZO-1 J Biol Chem 2005 280 3747 3756 10.1074/jbc.M411365200 15548514

[B56] Itoh M Furuse M Morita K Kubota K Saitou M Tsukita S Direct binding of three tight junction-associated MAGUKs, ZO-1, ZO-2, and ZO-3, with the COOH termini of claudins J Cell Biol 1999 147 1351 1363 10.1083/jcb.147.6.1351 PMC2168087 10601346

[B57] Itoh M Nagafuchi A Yonemura S Kitani-Yasuda T Tsukita S The 220-kD protein colocalizing with cadherins in non-epithelial cells is identical to ZO-1, a tight junction-associated protein in epithelial cells: cDNA cloning and immunoelectron microscopy J Cell Biol 1993 121 491 502 10.1083/jcb.121.3.491 PMC2119563 8486731

[B58] Hartsock A Nelson WJ Adherens and tight junctions: structure, function and connections to the actin cytoskeleton Biochim Biophys Acta 2008 1778 660 669 10.1016/j.bbamem.2007.07.012 PMC2682436 17854762

[B59] van Amsterdam K van der Ende A Nutrients released by gastric epithelial cells enhance Helicobacter pylori growth Helicobacter 2004 9 614 621 10.1111/j.1083-4389.2004.00272.x 15610074

[B60] Tan S Tompkins LS Amieva MR Helicobacter pylori usurps cell polarity to turn the cell surface into a replicative niche PLoS Pathog 2009 5 e1000407 10.1371/journal.ppat.1000407 PMC2669173 19412339

[B61] Ito T Kobayashi D Uchida K Takemura T Nagaoka S Kobayashi I Yokoyama T Ishige I Ishige Y Ishida N Helicobacter pylori invades the gastric mucosa and translocates to the gastric lymph nodes Lab Invest 2008 88 664 681 10.1038/labinvest.2008.33 18475258

[B62] Aspholm M Olfat FO Norden J Sonden B Lundberg C Sjostrom R Altraja S Odenbreit S Haas R Wadstrom T SabA is the H. pylori hemagglutinin and is polymorphic in binding to sialylated glycans PLoS Pathog 2006 2 e110 10.1371/journal.ppat.0020110 PMC1626103 17121461

[B63] Noach LA Rolf TM Tytgat GN Electron microscopic study of association between Helicobacter pylori and gastric and duodenal mucosa J Clin Pathol 1994 47 699 704 10.1136/jcp.47.8.699 PMC502139 7962619

[B64] Necchi V Candusso ME Tava F Luinetti O Ventura U Fiocca R Ricci V Solcia E Intracellular, intercellular, and stromal invasion of gastric mucosa, preneoplastic lesions, and cancer by Helicobacter pylori Gastroenterology 2007 132 1009 1023 10.1053/j.gastro.2007.01.049 17383424

[B65] Terres AM Pajares JM Hopkins AM Murphy A Moran A Baird AW Kelleher D Helicobacter pylori disrupts epithelial barrier function in a process inhibited by protein kinase C activators Infect Immun 1998 66 2943 2950 10.1128/iai.66.6.2943-2950.1998 PMC108293 9596771

[B66] Suzuki K Kokai Y Sawada N Takakuwa R Kuwahara K Isogai E Isogai H Mori M SS1 Helicobacter pylori disrupts the paracellular barrier of the gastric mucosa and leads to neutrophilic gastritis in mice Virchows Arch 2002 440 318 324 10.1007/s004280100430 11889604

[B67] Lytton SD Fischer W Nagel W Haas R Beck FX Production of ammonium by Helicobacter pylori mediates occludin processing and disruption of tight junctions in Caco-2 cells Microbiology 2005 151 3267 3276 10.1099/mic.0.28049-0 16207910

[B68] Pelicic V Reyrat JM Sartori L Pagliaccia C Rappuoli R Telford JL Montecucco C Papini E Helicobacter pylori VacA cytotoxin associated with the bacteria increases epithelial permeability independently of its vacuolating activity Microbiology 1999 145 Pt 8 2043 2050 10.1099/13500872-145-8-2043 10463170

[B69] Cohen D Brennwald PJ Rodriguez-Boulan E Musch A Mammalian PAR-1 determines epithelial lumen polarity by organizing the microtubule cytoskeleton J Cell Biol 2004 164 717 727 10.1083/jcb.200308104 PMC2172160 14981097

[B70] Zeaiter Z Cohen D Musch A Bagnoli F Covacci A Stein M Analysis of detergent-resistant membranes of Helicobacter pylori infected gastric adenocarcinoma cells reveals a role for MARK2/Par1b in CagA-mediated disruption of cellular polarity Cell Microbiol 2008 10 781 794 10.1111/j.1462-5822.2007.01084.x 18005242

[B71] Drewes G Ebneth A Preuss U Mandelkow EM Mandelkow E MARK, a novel family of protein kinases that phosphorylate microtubule-associated proteins and trigger microtubule disruption Cell 1997 89 297 308 10.1016/s0092-8674(00)80208-1 9108484

[B72] Lu H Murata-Kamiya N Saito Y Hatakeyama M Role of partitioning-defective 1/microtubule affinity-regulating kinases in the morphogenetic activity of Helicobacter pylori CagA J Biol Chem 2009 284 23024 23036 10.1074/jbc.M109.001008 PMC2755709 19553659

[B73] Ren S Higashi H Lu H Azuma T Hatakeyama M Structural basis and functional consequence of Helicobacter pylori CagA multimerization in cells J Biol Chem 2006 281 32344 32352 10.1074/jbc.M606172200 16954210

[B74] Ne Sbreve Ic D Miller MC Quinkert ZT Stein M Chait BT Stebbins CE Helicobacter pylori CagA inhibits PAR1-MARK family kinases by mimicking host substrates Nat Struct Mol Biol 2009 10.1038/nsmb.1705 PMC3006182 19966800

[B75] Lu HS Saito Y Umeda M Murata-Kamiya N Zhang HM Higashi H Hatakeyama M Structural and functional diversity in the PAR1b/MARK2-binding region of Helicobacter pylori CagA Cancer Sci 2008 99 2004 2011 10.1111/j.1349-7006.2008.00950.x PMC11158609 19016760

[B76] Papini E Satin B Norais N de Bernard M Telford JL Rappuoli R Montecucco C Selective increase of the permeability of polarized epithelial cell monolayers by Helicobacter pylori vacuolating toxin J Clin Invest 1998 102 813 820 10.1172/JCI2764 PMC508944 9710450

[B77] Crabtree JE Ferrero RL Kusters JG The mouse colonizing Helicobacter pylori strain SS1 may lack a functional cag pathogenicity island Helicobacter 2002 7 139 140 author reply 140-131 10.1046/j.1083-4389.2002.00071.x 11966874

[B78] Lapointe TK O'Connor PM Jones NL Menard D Buret AG Interleukin-1 receptor phosphorylation activates Rho kinase to disrupt human gastric tight junctional claudin-4 during Helicobacter pylori infection Cell Microbiol 2010 12 692 703 10.1111/j.1462-5822.2010.01429.x 20070312

[B79] Turner JR Rill BK Carlson SL Carnes D Kerner R Mrsny RJ Madara JL Physiological regulation of epithelial tight junctions is associated with myosin light-chain phosphorylation Am J Physiol 1997 273 C1378 1385 10.1152/ajpcell.1997.273.4.C1378 9357784

[B80] Zolotarevsky Y Hecht G Koutsouris A Gonzalez DE Quan C Tom J Mrsny RJ Turner JR A membrane-permeant peptide that inhibits MLC kinase restores barrier function in in vitro models of intestinal disease Gastroenterology 2002 123 163 172 10.1053/gast.2002.34235 12105845

[B81] Turner JR Angle JM Black ED Joyal JL Sacks DB Madara JL PKC-dependent regulation of transepithelial resistance: roles of MLC and MLC kinase Am J Physiol 1999 277 C554 562 10.1152/ajpcell.1999.277.3.C554 10484342

[B82] Chen Y Merzdorf C Paul DL Goodenough DA COOH terminus of occludin is required for tight junction barrier function in early Xenopus embryos J Cell Biol 1997 138 891 899 10.1083/jcb.138.4.891 PMC2138038 9265654

[B83] Saito Y Murata-Kamiya N Hirayama T Ohba Y Hatakeyama M Conversion of Helicobacter pylori CagA from senescence inducer to oncogenic driver through polarity-dependent regulation of p21 J Exp Med 2010 207 2157 2174 10.1084/jem.20100602 PMC2947069 20855497

[B84] Pertz O Bozic D Koch AW Fauser C Brancaccio A Engel J A new crystal structure, Ca2+ dependence and mutational analysis reveal molecular details of E-cadherin homoassociation Embo J 1999 18 1738 1747 10.1093/emboj/18.7.1738 PMC1171260 10202138

[B85] Gumbiner B Stevenson B Grimaldi A The role of the cell adhesion molecule uvomorulin in the formation and maintenance of the epithelial junctional complex J Cell Biol 1988 107 1575 1587 10.1083/jcb.107.4.1575 PMC2115263 3049625

[B86] Bershadsky A Magic touch: how does cell-cell adhesion trigger actin assembly? Trends Cell Biol 2004 14 589 593 10.1016/j.tcb.2004.09.009 15519846

[B87] Yonemura S Wada Y Watanabe T Nagafuchi A Shibata M alpha-Catenin as a tension transducer that induces adherens junction development Nat Cell Biol 2010 12 533 542 10.1038/ncb2055 20453849

[B88] Reynolds AB Daniel J McCrea PD Wheelock MJ Wu J Zhang Z Identification of a new catenin: the tyrosine kinase substrate p120cas associates with E-cadherin complexes Mol Cell Biol 1994 14 8333 8342 10.1128/mcb.14.12.8333-8342.1994 PMC359372 7526156

[B89] Yamada S Pokutta S Drees F Weis WI Nelson WJ Deconstructing the cadherin-catenin-actin complex Cell 2005 123 889 901 10.1016/j.cell.2005.09.020 PMC3368712 16325582

[B90] Drees F Pokutta S Yamada S Nelson WJ Weis WI Alpha-catenin is a molecular switch that binds E-cadherin-beta-catenin and regulates actin-filament assembly Cell 2005 123 903 915 10.1016/j.cell.2005.09.021 PMC3369825 16325583

[B91] Abe K Takeichi M EPLIN mediates linkage of the cadherin catenin complex to F-actin and stabilizes the circumferential actin belt Proc Natl Acad Sci USA 2008 105 13 19 10.1073/pnas.0710504105 PMC2224173 18093941

[B92] Chan AO Lam SK Wong BC Wong WM Yuen MF Yeung YH Hui WM Rashid A Kwong YL Promoter methylation of E-cadherin gene in gastric mucosa associated with Helicobacter pylori infection and in gastric cancer Gut 2003 52 502 506 10.1136/gut.52.4.502 PMC1773595 12631658

[B93] Leung WK Man EP Yu J Go MY To KF Yamaoka Y Cheng VY Ng EK Sung JJ Effects of Helicobacter pylori eradication on methylation status of E-cadherin gene in noncancerous stomach Clin Cancer Res 2006 12 3216 3221 10.1158/1078-0432.CCR-05-2442 16707623

[B94] Perri F Cotugno R Piepoli A Merla A Quitadamo M Gentile A Pilotto A Annese V Andriulli A Aberrant DNA methylation in non-neoplastic gastric mucosa of H. Pylori infected patients and effect of eradication Am J Gastroenterol 2007 102 1361 1371 10.1111/j.1572-0241.2007.01284.x 17509026

[B95] Chan AO Peng JZ Lam SK Lai KC Yuen MF Cheung HK Kwong YL Rashid A Chan CK Wong BC Eradication of Helicobacter pylori infection reverses E-cadherin promoter hypermethylation Gut 2006 55 463 468 10.1136/gut.2005.077776 PMC1856151 16428266

[B96] Conlin VS Curtis SB Zhao Y Moore ED Smith VC Meloche RM Finlay BB Buchan AM Helicobacter pylori infection targets adherens junction regulatory proteins and results in increased rates of migration in human gastric epithelial cells Infect Immun 2004 72 5181 5192 10.1128/IAI.72.9.5181-5192.2004 PMC517469 15322013

[B97] Weydig C Starzinski-Powitz A Carra G Lower J Wessler S CagA-independent disruption of adherence junction complexes involves E-cadherin shedding and implies multiple steps in Helicobacter pylori pathogenicity Exp Cell Res 2007 313 3459 3471 10.1016/j.yexcr.2007.07.015 17692843

[B98] Ogden SR Wroblewski LE Weydig C Romero-Gallo J O'Brien DP Israel DA Krishna US Fingleton B Reynolds AB Wessler S Peek RM Jr p120 and Kaiso regulate Helicobacter pylori-induced expression of matrix metalloproteinase-7 Mol Biol Cell 2008 19 4110 4121 10.1091/mbc.E08-03-0283 PMC2555941 18653469

[B99] Oliveira MJ Costa AM Costa AC Ferreira RM Sampaio P Machado JC Seruca R Mareel M Figueiredo C CagA associates with c-Met, E-cadherin, and p120-catenin in a multiproteic complex that suppresses Helicobacter pylori-induced cell-invasive phenotype J Infect Dis 2009 200 745 755 10.1086/604727 19604117

[B100] Suzuki M Mimuro H Kiga K Fukumatsu M Ishijima N Morikawa H Nagai S Koyasu S Gilman RH Kersulyte D Helicobacter pylori CagA phosphorylation-independent function in epithelial proliferation and inflammation Cell Host Microbe 2009 5 23 34 10.1016/j.chom.2008.11.010 19154985

[B101] Tolwinski NS Wieschaus E Rethinking WNT signaling Trends Genet 2004 20 177 181 10.1016/j.tig.2004.02.003 15041171

[B102] Tsukashita S Kushima R Bamba M Nakamura E Mukaisho K Sugihara H Hattori T Beta-catenin expression in intramucosal neoplastic lesions of the stomach. Comparative analysis of adenoma/dysplasia, adenocarcinoma and signet-ring cell carcinoma Oncology 2003 64 251 258 10.1159/000069310 12697966

[B103] Nakayama M Hisatsune J Yamasaki E Isomoto H Kurazono H Hatakeyama M Azuma T Yamaoka Y Yahiro K Moss J Hirayama T Helicobacter pylori VacA-induced inhibition of GSK3 through the PI3K/Akt signaling pathway J Biol Chem 2009 284 1612 1619 10.1074/jbc.M806981200 PMC2615499 18996844

[B104] Kurashima Y Murata-Kamiya N Kikuchi K Higashi H Azuma T Kondo S Hatakeyama M Deregulation of beta-catenin signal by Helicobacter pylori CagA requires the CagA-multimerization sequence Int J Cancer 2008 122 823 831 10.1002/ijc.23190 17960618

[B105] Pelz C Steininger S Weiss C Coscia F Vogelmann R A novel inhibitory domain of Helicobacter pylori protein CagA reduces CagA effects on host cell biology J Biol Chem 2011 286 8999 9008 10.1074/jbc.M110.166504 PMC3059056 21212271

[B106] Franco AT Johnston E Krishna U Yamaoka Y Israel DA Nagy TA Wroblewski LE Piazuelo MB Correa P Peek RM Jr Regulation of gastric carcinogenesis by Helicobacter pylori virulence factors Cancer Res 2008 68 379 387 10.1158/0008-5472.CAN-07-0824 PMC3114460 18199531

[B107] Hoy B Lower M Weydig C Carra G Tegtmeyer N Geppert T Schroder P Sewald N Backert S Schneider G Wessler S Helicobacter pylori HtrA is a new secreted virulence factor that cleaves E-cadherin to disrupt intercellular adhesion EMBO Rep 2010 11 798 804 10.1038/embor.2010.114 PMC2948180 20814423

[B108] Bebb JR Leach L Zaitoun A Hand N Letley DP Thomas R Atherton JC Effects of Helicobacter pylori on the cadherin-catenin complex J Clin Pathol 2006 59 1261 1266 10.1136/jcp.2006.036772 PMC1860537 16679349

[B109] Schirrmeister W Gnad T Wex T Higashiyama S Wolke C Naumann M Lendeckel U Ectodomain shedding of E-cadherin and c-Met is induced by Helicobacter pylori infection Exp Cell Res 2009 315 3500 3508 10.1016/j.yexcr.2009.07.029 19665015

[B110] Mayerle J Friess H Buchler MW Schnekenburger J Weiss FU Zimmer KP Domschke W Lerch MM Up-regulation, nuclear import, and tumor growth stimulation of the adhesion protein p120 in pancreatic cancer Gastroenterology 2003 124 949 960 10.1053/gast.2003.50142 12671892

[B111] Wijnhoven BP Pignatelli M Dinjens WN Tilanus HW Reduced p120ctn expression correlates with poor survival in patients with adenocarcinoma of the gastroesophageal junction J Surg Oncol 2005 92 116 123 10.1002/jso.20344 16231374

[B112] Sarrio D Moreno-Bueno G Sanchez-Estevez C Banon-Rodriguez I Hernandez-Cortes G Hardisson D Palacios J Expression of cadherins and catenins correlates with distinct histologic types of ovarian carcinomas Hum Pathol 2006 37 1042 1049 10.1016/j.humpath.2006.03.003 16867867

